# BAP1 loss impairs IFN-γ signaling and enhances NK cell-mediated cytotoxicity in myeloid leukemia

**DOI:** 10.1007/s00262-026-04453-5

**Published:** 2026-06-15

**Authors:** Chiara Badami, Erna Islamagic, Linnea Blomén, Frida Svensson, Theebiga Kathirkamanathan, Kristoffer Hellstrand, Elin Bernson, Fredrik B. Thorén

**Affiliations:** 1 TIMM Laboratory at Sahlgrenska Center for Cancer Research, University of Gothenburg, Box 450, 405 30 Gothenburg, Sweden; 2 Department of Medical Biochemistry and Cell Biology, Institute of Biomedicine, Sahlgrenska Academy, University of Gothenburg, Gothenburg, Sweden; 3 Department of Infectious Diseases, Institute of Biomedicine, Sahlgrenska Academy, University of Gothenburg, Gothenburg, Sweden; 4 Department of Obstetrics and Gynecology, Institute of Clinical Sciences, Sahlgrenska Academy, University of Gothenburg, Gothenburg, Sweden

**Keywords:** NK cells, CRISPR/Cas9, *BAP1*, IFN-γ, HLA-I

## Abstract

Natural killer (NK) cells recognize and eliminate malignant cells through multiple receptor–ligand interactions. To uncover genetic determinants of the susceptibility of myeloid leukemia cells to NK cell cytotoxicity, we analyzed several genome-wide CRISPR screens. Among recurrent hits, the *BRCA1-associated protein 1* (*BAP1*) gene emerged as a key factor protecting K562 leukemic cells from NK cell-mediated killing. Using *BAP1* knockout (KO) models, we found that loss of BAP1 alone did not alter NK cell sensitivity. However, upon interferon-γ (IFN-γ) stimulation, *BAP1* KO K562 cells exhibited reduced HLA class I induction, triggered enhanced NK cell degranulation, and showed increased sensitivity to NK cell-mediated cytotoxicity compared with wild-type cells. Further experiments revealed that BAP1-deficient cells displayed reduced expression of the IFN-γ receptor 1 (IFN-γ-R1). *BAP1* knockdown across multiple myeloid leukemia cell lines selectively decreased HLA-E and IFN-γ-R1 expression in *ASXL1*-mutant backgrounds. These findings suggest that BAP1 may contribute to the regulation of IFN-γ responsiveness and immune evasion in myeloid leukemia.

**Supplementary Information:**

The online version contains supplementary material available at 10.1007/s00262-026-04453-5.

## Introduction

NK cells are immune effector cells with an inherent capacity to kill virus-infected cells and malignant cells. NK cell cytotoxicity is tightly regulated through a balance of activating and inhibitory receptors that interact with cognate ligands on target cells. Activating receptors are linked to adaptor proteins containing immunoreceptor tyrosine-based activation motifs (ITAMs), which upon phosphorylation trigger activating signals. Major activating receptors are CD16, NKG2D, and the natural cytotoxicity receptors (NCRs) NKp46, NKp44, and NKp30 [1, 2]. On the other hand, inhibitory receptors contain immunoreceptor tyrosine-based inhibitory motifs (ITIMs). The main NK inhibitory receptors comprise the human leucocyte antigen (HLA)-specific killer immunoglobulin-like receptors (KIRs) and the CD94/NKG2A heterodimer [2] that recognizes non-classical HLA-E molecules. HLA-encoding genes have interferon (IFN)-responsive elements [3] and are upregulated in the presence of IFN type I (IFN-α/β) and type II (IFN-γ) [4, 5].

NK cells play a fundamental role in multiple types of cancers due to their ability to recognize malignant cells that downregulate HLA class I expression without the need of specific neoantigen presentation. NK cells are implicated in defense against multiple malignancies, including myeloid leukemias. Acute myeloid leukemia (AML) is a heterogeneous disease typically characterized by the accumulation of immature myeloid cells in bone marrow [6]. Additional sex combs-like 1 (ASXL1) is a recurrently mutated epigenetic regulator linked to poor prognosis in multiple myeloid malignancies, including AML [7–9], chronic myelomonocytic leukemia (CMML) [10–14], and chronic myeloid leukemia (CML) [15, 16]. ASXL1 contributes to epigenetic control through polycomb repressive complex 2 (PRC2)-mediated transcriptional repression [15, 17, 18] and by forming the polycomb repressive deubiquitinase (PR-DUB) complex with BRCA1-associated protein 1 (BAP1) [18]. Mutations in *ASXL1* commonly generate truncated proteins that disrupt interactions with PRC2, leading to de-repression of PRC2 target genes [17–19]. However, C-terminally truncated ASXL1 variants exhibit increased binding affinity for BAP1, resulting in enhanced deubiquitinating activity [20–22]. Within this PR-DUB complex, BAP1 removes ubiquitin from histone H2A, which promotes leukemogenesis [22, 23]. Thus, BAP1 has been proposed to be a potential therapeutic target in *ASXL1*-mutated myeloid disease [22–24].

Several studies have utilized genome-wide CRISPR screens to define structures that regulate cancer cell susceptibility to NK cells [25–32]. In this study, we reanalyzed publicly available CRISPR screen datasets and found that *BAP1* was recurrently found on the depleted side [25, 29–32] (Supplementary Table 1), suggesting that the loss of *BAP1* increases cancer cell susceptibility to NK cell killing. Deletion of *BAP1* in K562 cells resulted in increased sensitivity to NK cell cytotoxicity and induction of NK cell degranulation, but only in the presence of IFN-γ. Furthermore, *BAP1* knockout (KO) K562 cells displayed an impaired response to IFN-γ and thus an inability to upregulate IFN-γ-induced proteins, such as HLA class I, that trigger inhibitory receptors on NK cells. Interestingly, *BAP1* depletion resulted in lower HLA-E and IFN-γ-R1 expression in two different *ASXL1*-mutated cell lines but not in wild-type cell lines.

Taken together, these findings uncover a previously unrecognized role for BAP1 in modulating NK cell resistance and IFN-γ responsiveness in myeloid leukemia and provide a rationale for combining BAP1-targeted strategies with NK cell-based therapies.

## Materials and methods

### Antibodies

BAP1 (C-4, Santa Cruz, cat# sc-28383), secondary AF488-goat anti-mouse IgG (Invitrogen, cat# A11001), PE-Cy7-CD107a (BD, cat# 561348), BUV395-CD107a (BD, cat# 565113), PE-HLA-ABC (Thermo Fisher, cat# 12-9983-42), PE-Cy7-HLA-E (BioLegend, cat# 342608), PE-pSTAT1 (pY701) (BD, 562069), PE-total-STAT1 (BD, cat# 558537), PE-CD54 (BD, cat# 31625x), BV711-CD56 (BD, cat# 563169), and PE-CD119 (IFN-γ-R1) (BD, cat# 558934).

### Cell lines and NK cells

Myeloid leukemia cell lines were selected based on their relevance as commonly used experimental models, representation of distinct genetic backgrounds, and their suitability for efficient and reproducible *BAP1* knockdown. K562, PLB-985, OCI-AML2, and OCI-AML3 cell lines were cultured in Iscove’s modified Dulbecco’s medium (IMDM) (Gibco, cat# 21980,065) supplemented with HEPES, 10% fetal calf serum (FCS), 1% penicillin–streptomycin (PeSt) (Gibco, cat# 15140122), 1% L-glutamine (Gibco, cat# 25030024), and 1% sodium pyruvate (NaPyr) (Gibco, cat# 11360070) at 37 °C with 5% CO_2._ MEG-01 cell line was cultured in RPMI 1640 medium (Gibco, cat# 61870010) supplemented with GlutaMAX, HEPES, 10% fetal calf serum (FCS), 1% PeSt at 37 °C with 5% CO_2_.

Human peripheral blood mononuclear cells (PBMCs) were isolated from buffy coats (Sahlgrenska University Hospital). In brief, red blood cells were allowed to sediment with 2% dextran followed by density gradient centrifugation using Lymphoprep (STEMCELL Technologies, cat#07861). NK cells were isolated using the NK isolation kit (Miltenyi Biotech, cat#130-092-657) and activated overnight with 500U/ml IL-2.

For the 20-h co-incubation assay in which IFN-γ release was measured, polyclonally activated NK cells were generated as described elsewhere [32].

### *BAP1* gene deletion with CRISPR/Cas9 and knockdown using siRNA

*BAP1* KO cells were generated from Cas9-expressing K562 cells using a plasmid-based method. *BAP1* crRNAs were chosen among the GenScript pre-validated gRNA and Brunello library (crRNA1 TCAAATGGATCGAAGAGCGC crRNA2 TGGTGGATGATACGTCCGTG)*.*

The mEGFP-C1 (Addgene, cat# 54759) containing the previously inserted tracrRNA construct was used for *BAP1* crRNA insertion by BbsI-HF (NEB, cat# R3539L) cleavage and T4-DNA ligase (NEB, cat# M0202S) ligation. The recombinant plasmid was transformed into DH5α electroMAX cells (Invitrogen, cat# 11319019) and purified with the NucleoBond Xtra Midi EF (Macherey–Nagel, cat# 740410.50). The correct plasmid sequence was verified by Sanger sequencing (Eurofins Genomics). The plasmid containing the construct was transfected into K562 cells by electroporation with the Neon transfection system (Invitrogen, cat# 10090314). After 48 h, transfected K562 cells were single cell sorted by GFP expression using a BD FACSAria III. The gene KO was verified by Sanger sequencing.

Dicer-substrate small-interfering RNA (DsiRNA) targeting *BAP1* and a negative control DsiRNA (IDT) were delivered to myeloid leukemia cell lines by electroporation using the Neon Transfection System. Intranuclear staining and quantitative PCR (qPCR) were used to verify the reduction in BAP1 expression 48–72 h after transfection.

### Functional assays

In the dual-target cytotoxicity assay, wild-type (WT) K562 cells were pre-stained with cell trace Violet (Invitrogen, cat# C34557) and *BAP1* KO K562 cells were pre-stained with cell trace CFSE (Invitrogen, cat# C34554), incubated for 3 h with donor-isolated NK cells at an effector/target (ET) ratio of 1:1. The percentage of dead cells was determined using TO-PRO-3 (Invitrogen) staining. In degranulation assays, target cells were pre-stained with cell trace and incubated with NK cells for 3 h at an ET ratio 1:1 in the presence of an anti-CD107a antibody with NK cells alone used as a control. In the indicated dual-target cytotoxicity and degranulation assays, cells were either untreated or pre-treated for 20 h with 50 ng/ml recombinant human IFN-gamma (IFN-γ) (R&D, cat# 285-IF-100/CF), and NK cells were activated overnight with 500 U/ml IL-2. With the same setting, the killing of WT cells was compared with that of negative control-transfected cells to determine whether they were equivalent (Supplementary Fig. 2a).

Data acquisition was performed on a 5-laser BD LSRFortessa (BD Biosciences) or a 3-laser CytoFlex (Beckman Coulter). Data analysis was performed using FlowJo™ v10.9 and v10.10 Software (BD Biosciences). Gating strategies can be found in Supplementary Fig. 5a, b.

NK cell-derived IFN-γ form was detected by the enzyme-linked immunosorbent assay (ELISA) using the human IFN-gamma DuoSet ELISAs (R&D Systems, cat# DY285) according to the kit protocol and analyzed in the FLUOstar Omega Microplate Reader.

### Expression analysis with flow cytometry and qRT-PCR

Surface staining of K562 cells was performed in E-buffer (buffered saline supplemented with 0,1% EDTA and 0,5% BSA) for 30 min at 4 °C. Intranuclear staining and phosphoflow were performed with BD Cytofix buffer (cat# 554655), BD Phosflow™ Perm Buffer III (cat# 558050), and BD Stain Buffer (cat554656) according to the manufacturer’s protocol. Each stained sample was compared with the corresponding unstained or stained with only secondary antibody control (representative plots are included in Fig. 3d and Supplementary Fig. 5d for IFN-γ-R1 and pSTAT1, respectively). Gating strategies can be found in Supplementary Fig. 5c.

For transcript analysis, RNA was isolated with RNeasy Plus Mini Kit (QIAGEN) and RNA concentration was measured with a Nanodrop. Complementary DNA was generated with the TATAA GrandScript cDNA Synthesis Kit (TATAA Biocenter) in a Veriti Thermal Cycler (Applied Biosystems), and qPCR was performed with the TATAA SYBR GrandMaster Mix (TATAA Biocenter) in CFX384 Touch Real-Time PCR Detection System (Bio-Rad) following manufacturer’s recommendations. Gene expression was compared to the expression of housekeeping genes (*GAPDH* and *TBP*), and relative expression was obtained by 2^−ΔCt^. The primers used are listed in Supplementary Table 2.

### Mass spectrometry

Relative quantitative mass spectrometry using isobaric labeling (TMT-MS) was performed at the Proteomics Core Facility (PCF) at the University of Gothenburg to compare protein abundances in WT and *BAP1* KO K562 with or without IFN-γ pre-treatment, in four replicates per condition from four independent cultures. Proteins were digested with trypsin, and the resulting peptides were labeled with tandem mass tag (TMT) reagents, assigning a unique isobaric tag to each sample. Labeled samples were then pooled at equal peptide amounts and analyzed by nano-liquid chromatography mass spectrometry (nanoLC-MS/MS). Peptides were ionized, isolated by mass-to-change ratio, and fragmented for peptide identification. Upon fragmentation, TMT reporter ions were released and measured. Relative protein abundance was determined from the intensity ratios of reporter ions across samples.

### Statistical analysis

Statistical analysis was performed using GraphPad Prism (version 10 and 11) and R Studio. *T* test or one-way ANOVA followed by Šidák’s multiple comparison test was used to test significance in functional assays, to compare median fluorescence intensities (MFIs), and relative expression. Unequal variance unpaired *T* test was used for comparing protein abundances in proteomic analysis. *P* values < 0.05 were considered statistically significant.

## Results

### *BAP1* depletion increases K562 cell susceptibility to NK cells in an IFN-γ-dependent manner

**Fig. 1:**
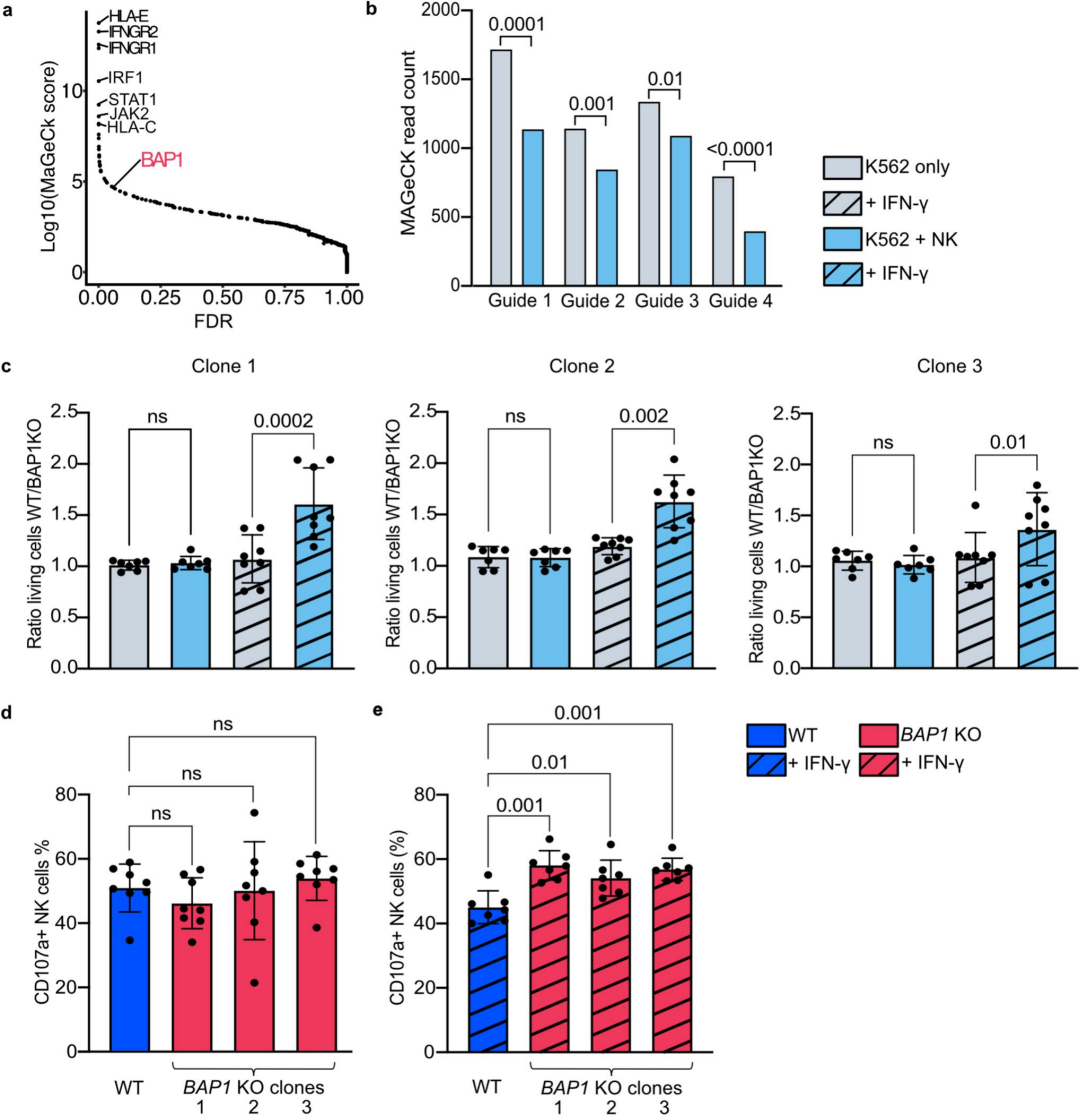
*BAP1* KO reduces K562 cell protection to NK cells in the presence of IFN-γ. **a** Top-depleted genes in Kristenson et al. [32] CRISPR screen. **b** Normalized read counts from four different guides toward *BAP1* in Kristenson et al. CRISPR screen [32]. **c** Ratio of living WT/*BAP1* KO cells with or without NK challenging from dual-target 3-h cytotoxicity assay with untreated or IFN-γ-treated WT K562 cells and three different *BAP1* KO clones (n = 7 untreated, *n* = 8 IFN-γ treated). **d**, **e** NK cell degranulation toward untreated (**d**) and IFN-γ-treated (**e**) WT K562 cells versus three *BAP1* KO clones (*n* = 8 untreated, *n* = 7 IFN-γ treated) co-incubated for 3 h. One-way ANOVA followed by Šidák’s multiple comparison test was used in c, d, and e. ns = not significant. Error bars represent SD

In an attempt to identify novel regulatory elements of NK cell susceptibility, we analyzed publicly available CRISPR screen datasets performed by us and others (Supplementary Table 1) [25–32]. In screens using primary NK cells, target cells that had lost genes driving IFN-γ signaling and antigen presentation were consistently found on the depleted side, reflecting the fact that downregulated HLA class I expression sensitizes cancer cells to NK cell cytotoxicity. Interestingly, *BAP1* was also recurrently present among depleted genes [25, 29–32]. Accordingly, all four *BAP1*-directed guide RNAs in the Brunello library were associated with high susceptibility to NK cell cytotoxicity in our CRISPR screen (Fig. 1a, b) [32], suggesting that intact BAP1 expression protects leukemic cells against NK cell cytotoxicity.

*BAP1* mutations are rare in leukemia [33, 34], although they have been reported in specific subtypes [35]. In contrast, the gene encoding its binding partner, ASXL1, is frequently mutated across myeloid malignancies [7–16]. Given the functional interaction between BAP1 and ASXL1, we sought to investigate the role of BAP1 in modulating NK cell responses in myeloid leukemia, including *ASXL1*-mutated (*ASXL1*-MT) contexts. To this end, we used CRISPR/Cas9 technology and single cell sorting to establish monoclonal *BAP1* KO variants in K562 cells (Supplementary Fig. 1a).

We compared the sensitivity of *BAP1* KO and WT K562 cells to NK cell cytotoxicity using a dual-target cytotoxicity assay. Differentially labeled *BAP1* KO and WT K562 target cells were exposed to overnight-activated NK cells. No difference between WT and *BAP1* KO K562 cells in NK cell susceptibility was observed in these 3h assays (Fig. 1c and Supplementary Fig. 1b). However, the original CRISPR screen was performed after a 20-h co-incubation with polyclonally activated and expanded NK cells [32]. As mentioned above, genes related to IFN-γ signaling were associated with target cell survival in CRISPR screens, suggesting a key role for NK cell-derived IFN-γ in the assays. We speculated that insufficient IFN-γ was produced during the 3h assay and that pre-treatment of K562 cells with IFN-γ could re-capitulate the CRISPR screen findings. As expected, we found IFN-γ release after the 20-h co-incubation of polyclonally activated NK cells and K562 cells to be significantly higher than the minute levels found in supernatants recovered from the 3-h assay (Supplementary Fig. 1c).

These results prompted us to pre-treat K562 cells with IFN-γ. In accordance with the screen results, this pre-treatment resulted in predominant killing of BAP1-deficient cells (Fig. 1c and Supplementary Fig. 1b). Accordingly, no difference in NK cell degranulation was observed after short-term experiments without pre-stimulation against *BAP1* KO and WT K562 cells (Fig. 1d), whereas a significant increase in NK degranulation was observed against *BAP1* KO cells versus WT cells after pre-stimulation with IFN-γ (Fig. 1e). Taken together, these results imply that the absence of BAP1 sensitizes target cells to NK cell-mediated killing in the presence of IFN-γ.

### *BAP1* deletion impairs K562 cell response to IFN-γ and antigen presentation

**Fig. 2:**
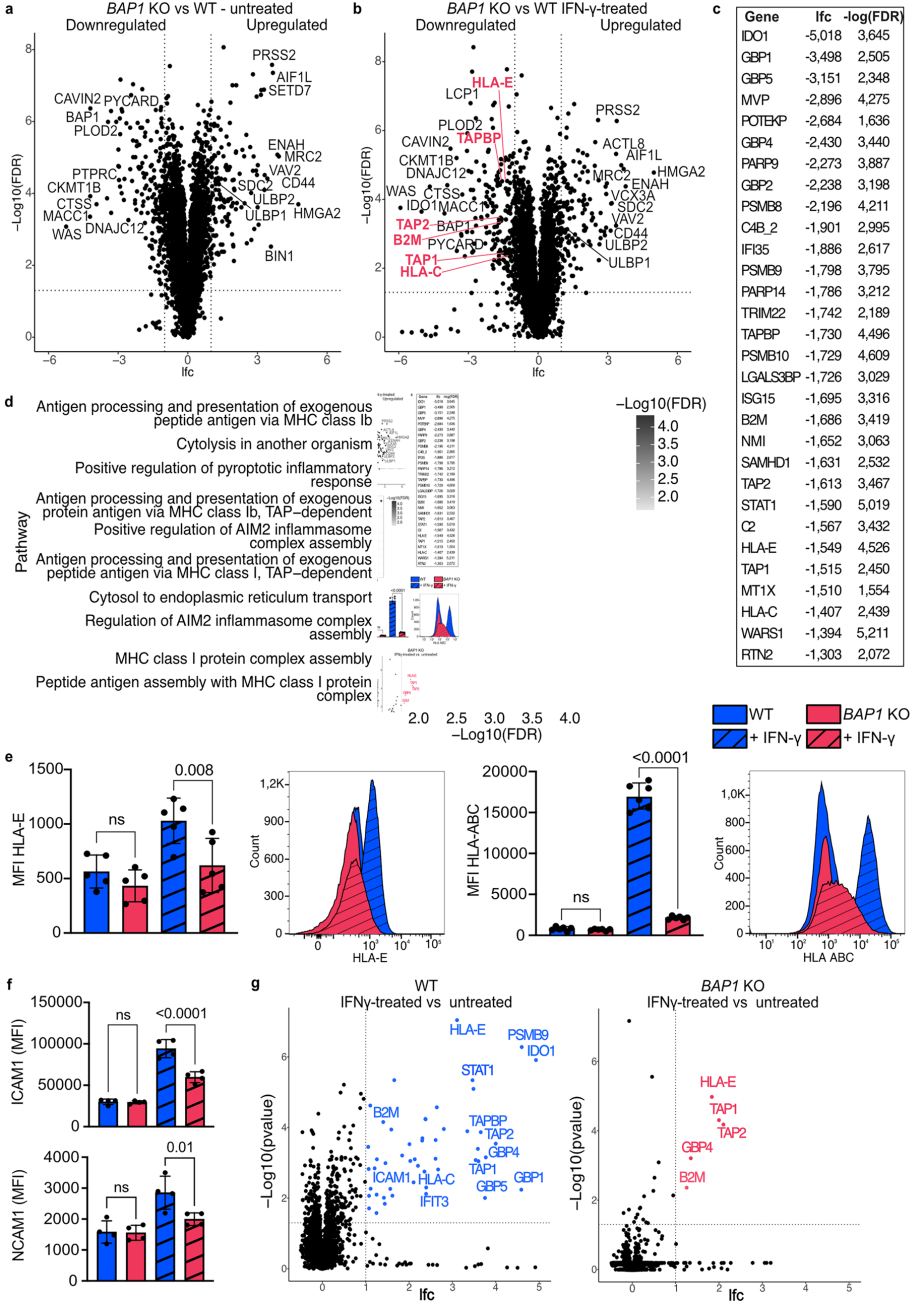
*BAP1* KO impairs K562 cell response to IFN-γ and antigen presentation pathway. **a**, **b** Proteome analysis showing differentially regulated proteins of *BAP1* KO versus WT K562 cells in an untreated (**a**) and IFN-γ-treated (**b**) condition. **c** Top 30 downregulated proteins in *BAP1* KO versus WT K562 cells after IFN-γ treatment but not in the untreated condition ranked by log fold change (lfc). **d** Gene Ontology top 10 biological process pathway analysis of the downregulated proteins in *BAP1* KO versus WT K562 cells following IFN-γ treatment that were not downregulated in the untreated condition. **e**, **f** Staining for IFN-γ-induced HLA class I (**e**) and CAMs (**f**) in *BAP1* KO and WT K562 cells without and with IFN-γ treatment (HLA-I *n* = 5, CAMs *n* = 4). **g** Proteome analysis of *BAP1* KO and WT K562 cells showing upregulated proteins upon IFN-γ treatment. *T* test for different variances and FDR was used for statistical analysis in a, b, c, and g. One-way ANOVA followed by Šidák’s multiple comparison test was used in e and f. ns = not significant. Error bars represent SD

The results above suggest that *BAP1* KO cells respond differently to IFN-γ stimulation. IFN-γ binds to IFN receptors (IFN-γ-R) that activate the JAK/STAT pathway, resulting in the downstream transcription and translation of various genes and proteins. With the aim to clarify to what extent *BAP1* deletion affected the cellular proteome, tandem mass tag mass spectrometry (TMT-MS) was performed to compare the proteome of *BAP1* KO cells and WT cells in the presence or absence of IFN-γ pre-treatment. The proteomic profiles of *BAP1* knockout and wild-type samples differed markedly in both untreated (Fig. 2a) and IFN-γ-treated (Fig. 2b) conditions, highlighting the broad impact of BAP1 loss on cancer cells. Notably, a substantial number of proteins induced by IFN-γ were significantly downregulated in *BAP1* KO cells as compared to WT cells (Fig. 2b, c). Gene Ontology biological process (Fig. 2d), molecular function (Supplementary Fig. 2b), and KEGG pathway (Supplementary Fig. 2c) analyses of proteins downregulated in *BAP1* KO—but not WT—cells following IFN-γ treatment, indicated that BAP1 loss affected the expression of proteins involved in antigen presentation pathways. Among these differentially expressed proteins were HLA class I molecules, which serve as ligands to the inhibitory NK cell receptors, KIRs and NKG2A/CD94. To validate these findings, we assessed HLA class I expression by flow cytometry and qPCR (Fig. 2e and Supplementary Fig. 1d, e) along with other IFN-γ*-*induced proteins relevant to NK–tumor cell interactions, including the cell adhesion molecules (CAMs), CD54 (ICAM1), and CD56 (NCAM1) (Fig. 2f). In WT K562 cells, IFN-γ treatment induced a pronounced upregulation of these molecules. By contrast, *BAP1* KO cells exhibited an impaired response to IFN-γ treatment, consistent with the reduced induction of IFN-γ-responsive proteins observed in the proteomic analysis (Fig. 2g). The diminished upregulation of ICAM1 in *BAP1* KO cells (Fig. 2f, g) suggests decreased adhesion to NK cells. Furthermore, the proteomic profiling indicated an upregulation of the NKG2D ligands ULBP1/2 in *BAP1* knockout samples (Fig. 2b). However, flow cytometric analysis of ULBP1 and ULBP2/5/6 expression in BAP1-deficient K562 cells revealed only minor, nonsignificant differences (Supplementary Fig. 1f), suggesting that these alterations do not account for the increased NK cell sensitivity observed. Hence, the altered HLA class I expression appears to be the dominant factor driving the increased susceptibility to NK cell-mediated cytotoxicity.

Taken together, the data underline a previously unrecognized role of BAP1 in modulating IFN-γ responses. Indeed, the upregulation of proteins induced by IFN-γ such as HLA-I expression-related proteins and proteins belonging to the guanylate-binding protein family was severely compromised in *BAP1* KO cells (Fig. 2c, g).

### *BAP1* depletion affects upstream elements of the IFN-γ pathway

**Fig. 3:**
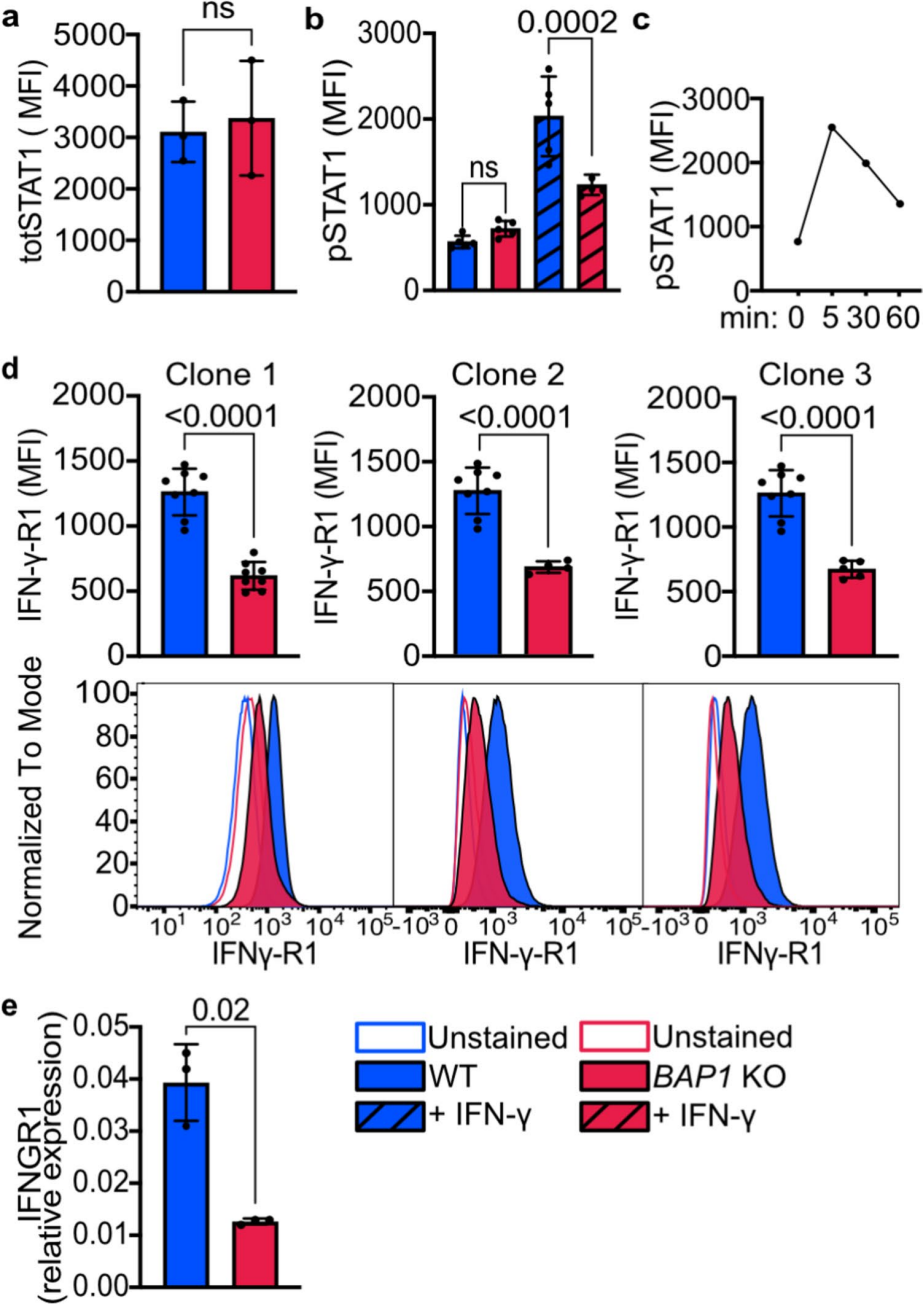
*BAP1* KO compromises the expression of the upstream elements of the IFN-γ-response pathway. **a** Total STAT1 staining without IFN-γ treatment of *BAP1* KO K562 versus WT cells (*n* = 3). **b** Phosphorylated STAT1 staining of *BAP1* KO K562 versus WT cells without and with IFN-γ treatment (*n* = 5). **c** STAT1 phosphorylation in untreated K562 cells and after 5 min, 30 min, or 1 h of IFN-γ treatment. **d** IFN-γ-R1 staining in three *BAP1* KO clones compared to WT K562 cells (WT and clone 1 *n* = 8, clone 2 *n* = 4, clone 3 *n* = 5). **e** *IFNGR1* transcript expression in *BAP1* KO K562 cells versus WT (*n* = 3). *T* test was used for statistical analysis in a, d, and e. One-way ANOVA followed by Šidák’s multiple comparison test was used in b. ns = not significant. Error bars represent SD

We next explored where along the IFN-γ signaling pathway BAP1 deficiency exerted its effect. Analysis of STAT1 expression and phosphorylation revealed that total STAT1 and basal pSTAT1 levels were unaffected by loss of BAP1 (Fig. 3a, b). In response to IFN-γ, STAT1 Y701 phosphorylation peaked after 5 min (Fig. 3c). *BAP1* KO cells consistently displayed significantly reduced pSTAT1 staining after IFN-γ treatment as compared to WT cells (Fig. 3b), pointing toward a proximal impairment in signaling. Accordingly, cell surface staining analysis and qPCR revealed that the expression of IFN-γ receptor 1 (IFN-γ-R1) was significantly lower in *BAP1* KO cells both at protein (Fig. 3d and Supplementary Fig. 1g) and transcript (Fig. 3e) levels.

### *BAP1* modulates antigen presentation and IFN-γ response in *ASXL1*-mutated myeloid leukemic cells

**Fig. 4:**
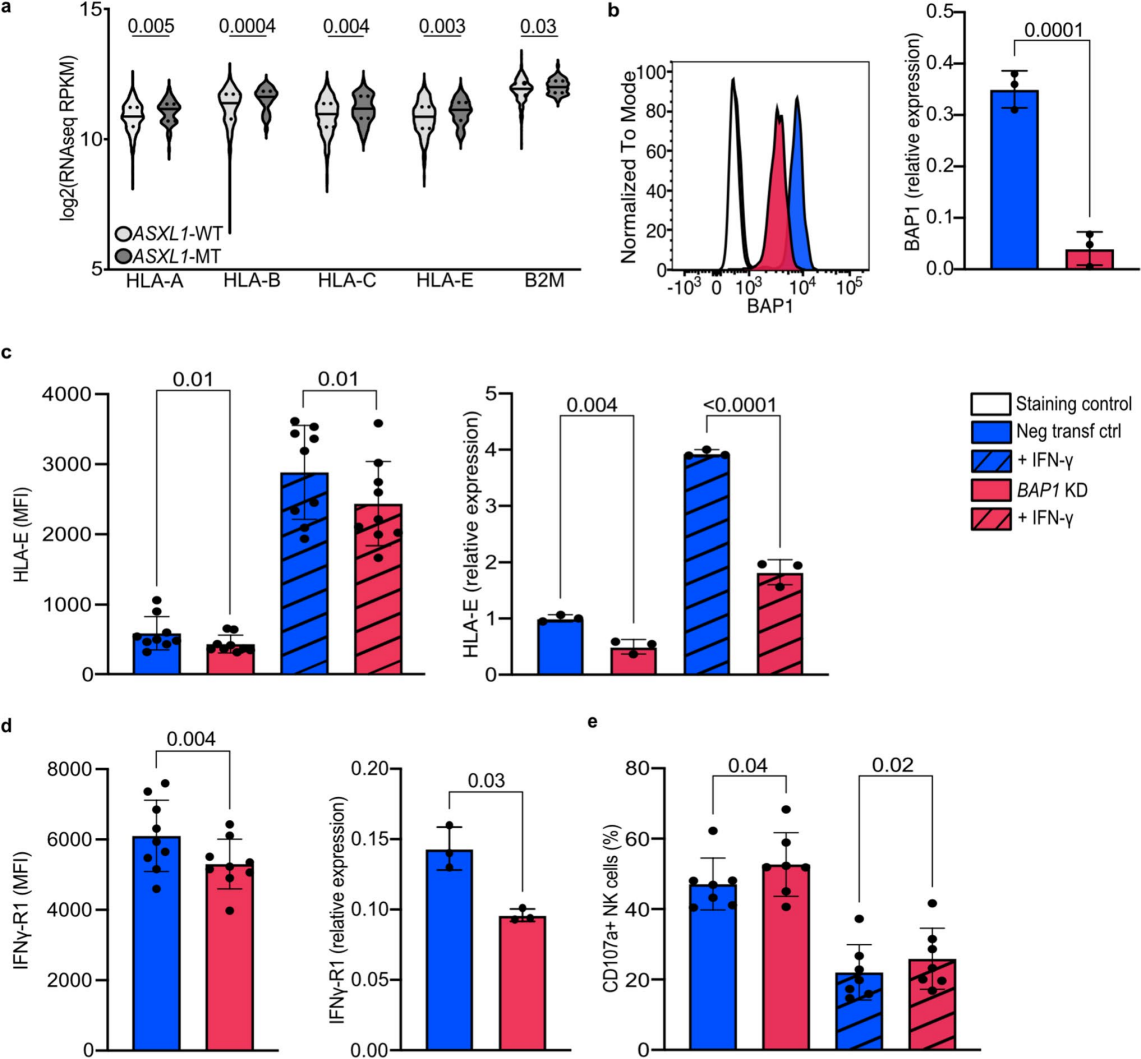
*BAP1* KD impairs antigen presentation and IFN-γ response in the *ASXL1*-mutated leukemia cell line MEG-01. **a** Expression of genes involved in IFN-γ response and antigen presentation in AML patients carrying an *ASXL1* mutation (*ASXL1*-MT) or not (*ASXL1*-WT), from the OHSU dataset (2022) [38]. **b** BAP1 intranuclear staining and transcript of negative control and *BAP1* KD MEG-01 cells (n = 3). **c** HLA-E surface and transcript expression of *BAP1* KD MEG-01 cells versus negative control-transfected cells (*n* = 9, *n* = 3). **d** IFN-γ-R1 surface staining and *IFNGR1* transcript expression of negative control and *BAP1* KD MEG-01 (*n* = 9, *n* = 3). **e** NK cell degranulation toward untreated and IFN-γ pre-treated *BAP1* KD and negative control MEG-01 cells (*n* = 7). Mann–Whitney *U* test and Benjamini–Hochberg were used for statistical analysis in a. *T* test was used in b and d. One-way ANOVA and Šidák’s multiple comparison test were used in c and e. Error bars represent SD

As mentioned, BAP1 forms a PR-DUB complex with ASXL1 [18]. K562 cells harbor a mutation in the *ASXL1* gene (Y591Y/X) [36, 37] that leads to the formation of a truncated ASXL1 protein. Such truncations have been demonstrated to enhance ASXL1 binding to BAP1 [21–23] and to entail reduced interaction with the PRC2 complex, which may affect expression levels of interferon-stimulated genes [17, 19]. For this reason, we investigated whether BAP1-dependent modulation of the IFN-γ response and antigen presentation was also related to the presence of truncated ASXL1. Exploring a publicly available dataset of bulk RNA sequencing data in AML patients with defined mutations [38], AML patients harboring *ASXL1* mutations showed increased mRNA levels of genes involved in IFN-γ response and antigen presentation as compared to *ASXL1* wild-type (*ASXL1*-WT) patients (Fig. 4a) supporting the hypothesis that the ASXL1-MT–BAP1 complex is implicated in antigen presentation and HLA class I expression. Notably, no difference in BAP1 mRNA expression was present between *ASXL1*-WT and mutated patients (Supplementary Fig. 2d). Interestingly, *NCR3LG1* encoding B7-H6 is expressed at lower levels in *ASXL1*-MT patients, which could be in line with poorer prognosis (Supplementary Fig. 2e).

We next performed *BAP1* knockdown (KD) using siRNA in the *ASXL1*-MT MEG-01 cell line and in myeloid leukemia *ASXL1*-WT cell lines PLB-985, OCI-AML2, and OCI-AML3 (Fig. 4b and Supplementary Fig. 3a). Consistent with observations in *ASXL1*-MT K562 cells, *BAP1* knockdown in MEG-01 cells resulted in a significant reduction in HLA-E and IFN-γ-R1 expression as compared to negative control-transfected cells (Fig. 4c, d). In line with this, *BAP1* depletion in MEG-01 cells triggered increased NK degranulation relative to control cells (Fig. 4e). In contrast, although a reduction in IFN-γ-R1 surface protein expression was also observed in *BAP1*-silenced OCI-AML2 cells (Supplementary Fig. 3c), none of the *ASXL1*-WT cell lines recapitulated the effects on HLA-E surface expression or NK degranulation (Supplementary Fig. 3b, d). BAP1 depletion was associated with reduced IFN-γ-induced HLA class I transcripts in most cell lines (Fig. 4c, Supplementary Figs. 1e, 3b and 4a–c), but the magnitude of this effect varied, and protein levels were not significantly reduced in the *ASXL1*-WT cell lines.

## Discussion

This study identifies BAP1 as a recurrent regulator of NK cell cytotoxicity across multiple CRISPR screens and uncovers a potential mechanistic link between BAP1–ASXL1 complex function, IFN-γ signaling, and NK cell resistance in myeloid leukemia. Our findings imply that the loss of BAP1, particularly in the context of *ASXL1*-truncating mutations, increases leukemic cell susceptibility to NK cell-mediated killing by disrupting interferon responses and antigen presentation pathways.

BAP1 is involved in multiple cellular processes, including chromatin remodeling and transcriptional regulation. Consistent with its pleiotropic role, proteomic profiling revealed broad changes in protein expression of myeloid leukemic cells following *BAP1* depletion. Among these, components of the antigen processing and presentation machinery were notably downregulated after IFN-γ treatment in *BAP1* KO cells as compared to WT cells, suggesting that BAP1 is required for an efficient transcriptional response to IFN-γ stimulation. This interpretation was further supported by flow cytometry data showing that the induction of HLA-E and HLA-ABC expression was strongly compromised in *BAP1* KO K562 cells after IFN-γ stimulation. Mechanistically, BAP1 loss resulted in reduced IFN-γ receptor expression and attenuated STAT1 phosphorylation, indicating that BAP1 integrity is necessary for efficient propagation of IFN-γ signaling. This impairment provides a plausible explanation for the observed sensitivity to NK cells and proposes BAP1 as a previously unrecognized regulator of interferon responsiveness in leukemic cells.

Notably, the functional consequences of altered IFN-γ responsiveness are likely shaped by the combined regulation of multiple NK cell ligands. While reduced HLA class I expression would be expected to enhance NK cell activation, we observed that BAP1-deficient cells also exhibited diminished induction of the adhesion molecule ICAM1. Given the role of ICAM1 in stabilizing NK–target cell interactions and promoting effective cytotoxic synapse formation, its reduced expression may limit NK cell engagement and partially offset the effects of decreased HLA-mediated inhibition.

Proteomic profiling suggested an upregulation of NKG2D ligands in BAP1-deficient cells; however, this was not corroborated by flow cytometric analysis of ULBP expression. Although subtle, these proteomic alterations may reflect additional mechanisms contributing to NK cell sensitization beyond the impaired IFN-γ-mediated upregulation of HLA class I and underscore that the net NK cell response reflects the integration of both inhibitory and activating signals, as well as adhesion-dependent interactions, rather than changes in any single ligand.

K562 leukemic cells harbor a common truncating mutation in the *ASXL1* gene [36, 37], which leads to a hyperfunctional BAP1–ASXL1 complex. Analysis of the OHSU AML dataset [38] revealed an increased expression of genes involved in IFN-γ response and antigen presentation in *ASXL1-*mutated cases. In addition to enhanced expression of inhibitory HLA class I molecules, the activating ligand B7-H6 was significantly reduced in patients carrying *ASXL1* mutations. Together, these findings may be consistent with an NK cell-evasive phenotype that could, at least in part, contribute to the increased aggressiveness of *ASXL1*-mutated AML. However, these observations are based on bulk RNA-sequence data and should be interpreted with caution. Gene expression signals reflect both malignant and non-malignant compartments and do not account for potential differences in blast faction or immune/stromal composition between *ASXL1*-MT and WT.

Although our analysis was limited to a small number of myeloid leukemia cell lines, reduced HLA-E and IFN-γ-R1 surface expression was observed only in the presence of *ASXL1* mutations. While this may suggest a context-dependent interaction between these epigenetic regulators, the limited dataset precludes firm conclusions. ASXL1 and BAP1 are both involved in modulating the activity of polycomb repressive complex 2 (PRC2), which mediates transcriptional silencing of target genes through H3K27 methylation. Among genes targeted by PRC2 are various genes involved in HLA class I presentation and the IFN-γ pathway [39, 40]. The *ASXL1-*truncating mutations are associated with disrupted PRC2 activity, reduced H3K27 methylation, and loss of PRC2-dependent gene repression [17, 19, 41], and previous studies have shown that BAP1 loss can increase EZH2 levels [39, 40, 42].

Together, these observations raise the possibility that BAP1 contributes to the regulation of IFN-γ-R1 and HLA class I expression through modulation of PRC2 activity. In *ASXL1*-mutated contexts, reduced BAP1 expression could potentially influence H3K27 methylation and thereby affect the expression of PRC2 target genes, including those involved in antigen presentation and IFN-γ signaling [39, 40]. However, this model remains speculative and requires further experimental validation.

In line with previous work by Wijdeven et al. [43], which identified BAP1 as a positive regulator of MHC class I independently of IFN-γ stimulation, we observed a similar effect in MEG-01 cells. In contrast, no clear effect was observed in unstimulated K562 cells, possibly due to their inherently low HLA class I expression.

Overall, our findings suggest a potential role for BAP1 in modulating IFN-γ responsiveness and NK cell interactions, with effects that may be influenced by the *ASXL1* mutational status. However, the mechanisms underlying IFN-γ-R1 regulation by BAP1 remain unresolved. Future studies will be needed to elucidate whether BAP1 deficiency directly affects *IFNGR1* chromatin accessibility or acts through alternative pathways. Moreover, experiments introducing *ASXL1* mutations into *ASXL1*-WT AML cell lines will be necessary to clarify the extent to which the observed phenotype is specifically attributed to *ASXL1* status.

The impact of BAP1 expression on IFN-γ responses may be relevant in vivo where IFN-γ signaling is frequently upregulated in AML and can contribute to therapy resistance [44] as well as in the context of hematopoietic stem cell transplantation (HSCT), where allogeneic NK and T cells may produce high levels of IFN-γ upon recognition of leukemic cells. IFN-γ-driven upregulation of HLA class I molecules can enhance antigen presentation and promote T cell-mediated recognition, but at the same time may limit NK cell activity through engagement of inhibitory receptors. In this context, impaired IFN-γ responsiveness, as observed upon BAP1 loss, may shift this balance by reducing HLA class I expression and thereby favoring NK cell-mediated cytotoxicity.

Modulation of leukemic cell sensitivity to NK cell—for example through reduced HLA-E expression—may represent a potential avenue to enhance anti-leukemic immunity, although this remains to be formally tested. More broadly, our findings highlight the importance of considering how leukemic cells, including those harboring *ASXL1* mutations, differentially respond to IFN-γ to shape their interaction with both NK and T cell compartments.

Taken together, our study supports a link between BAP1 function, interferon signaling, and immune recognition in myeloid leukemia and provides a rationale for further investigation of NK cell-based therapeutic strategies, particularly in settings where T cell responses may be limited or counterbalanced by HLA-mediated inhibition.

## Supplementary Information

Below is the link to the electronic supplementary material.

Supplementary file1 (DOCX 5382 KB)

## Data Availability

The data that support the findings of this study are available from the corresponding author upon reasonable request.

## References

[1] Quatrini L et al (2021) Human NK cells, their receptors and function. Eur J Immunol 51(7):1566–1579 33899224 10.1002/eji.202049028 PMC9292411

[2] Bryceson YT et al (2006) Activation, coactivation, and costimulation of resting human natural killer cells. Immunol Rev 214:73–91 17100877 10.1111/j.1600-065X.2006.00457.x PMC3845883

[3] Johnson DR, Pober JS (1994) HLA class I heavy-chain gene promoter elements mediating synergy between tumor necrosis factor and interferons. Mol Cell Biol 14(2):1322–1332 8289810 10.1128/mcb.14.2.1322-1332.1994 PMC358487

[4] Drew PD et al (1993) Regulation of MHC class I and beta 2-microglobulin gene expression in human neuronal cells. Factor binding to conserved cis-acting regulatory sequences correlates with expression of the genes. J Immunol 150(8 Pt 1):3300–3310 8468472

[5] Keskinen P et al (1997) Regulation of HLA class I and II expression by interferons and influenza A virus in human peripheral blood mononuclear cells. Immunology 91(3):421–429 9301532 10.1046/j.1365-2567.1997.00258.x PMC1364012

[6] Yamashita M et al (2020) Dysregulated haematopoietic stem cell behaviour in myeloid leukaemogenesis. Nat Rev Cancer 20(7):365–382 32415283 10.1038/s41568-020-0260-3 PMC7658795

[7] Schnittger S et al (2013) ASXL1 exon 12 mutations are frequent in AML with intermediate risk karyotype and are independently associated with an adverse outcome. Leukemia 27(1):82–91 23018865 10.1038/leu.2012.262

[8] Rocquain J et al (2010) Combined mutations of ASXL1, CBL, FLT3, IDH1, IDH2, JAK2, KRAS, NPM1, NRAS, RUNX1, TET2 and WT1 genes in myelodysplastic syndromes and acute myeloid leukemias. BMC Cancer 10:401 20678218 10.1186/1471-2407-10-401 PMC2923633

[9] Metzeler KH et al (2011) ASXL1 mutations identify a high-risk subgroup of older patients with primary cytogenetically normal AML within the ELN Favorable genetic category. Blood 118(26):6920–6929 22031865 10.1182/blood-2011-08-368225 PMC3245212

[10] Abdel-Wahab O et al (2011) Concomitant analysis of EZH2 and ASXL1 mutations in myelofibrosis, chronic myelomonocytic leukemia and blast-phase myeloproliferative neoplasms. Leukemia 25(7):1200–1202 21455215 10.1038/leu.2011.58 PMC4641450

[11] Patnaik MM et al (2013) Mayo prognostic model for WHO-defined chronic myelomonocytic leukemia: ASXL1 and spliceosome component mutations and outcomes. Leukemia 27(7):1504–1510 23531518 10.1038/leu.2013.88

[12] Itzykson R et al (2013) Prognostic score including gene mutations in chronic myelomonocytic leukemia. J Clin Oncol 31(19):2428–2436 23690417 10.1200/JCO.2012.47.3314

[13] Gelsi-Boyer V et al (2010) ASXL1 mutation is associated with poor prognosis and acute transformation in chronic myelomonocytic leukaemia. Br J Haematol 151(4):365–375 20880116 10.1111/j.1365-2141.2010.08381.x

[14] Gelsi-Boyer V et al (2012) Mutations in ASXL1 are associated with poor prognosis across the spectrum of malignant myeloid diseases. J Hematol Oncol 5:12 22436456 10.1186/1756-8722-5-12 PMC3355025

[15] Shanmuganathan N et al (2025) Impact of ASXL1 at diagnosis in patients with CML receiving frontline potent TKIs: high risk of kinase domain mutations. Blood 146(23):2821–2832 40896831 10.1182/blood.2025030259

[16] Schönfeld L et al (2022) ASXL1 mutations predict inferior molecular response to nilotinib treatment in chronic myeloid leukemia. Leukemia 36(9):2242–2249 35902731 10.1038/s41375-022-01648-4 PMC9417980

[17] Abdel-Wahab O et al (2012) ASXL1 mutations promote myeloid transformation through loss of PRC2-mediated gene repression. Cancer Cell 22(2):180–193 22897849 10.1016/j.ccr.2012.06.032 PMC3422511

[18] Scheuermann JC et al (7295) Histone H2A deubiquitinase activity of the Polycomb repressive complex PR-DUB. Nature 465(7295):243–247 20436459 10.1038/nature08966 PMC3182123

[19] Tamburri S et al (2020) Histone H2AK119 Mono-Ubiquitination Is Essential for Polycomb-Mediated Transcriptional Repression. Mol Cell 77(4):840-856.e5 31883952 10.1016/j.molcel.2019.11.021 PMC7033561

[20] Campagne A et al (2019) BAP1 complex promotes transcription by opposing PRC1-mediated H2A ubiquitylation. Nat Commun 10(1):348 30664650 10.1038/s41467-018-08255-x PMC6341105

[21] Balasubramani A et al (2015) Cancer-associated ASXL1 mutations may act as gain-of-function mutations of the ASXL1-BAP1 complex. Nat Commun 6:ASXL1 10.1038/ncomms8307 PMC4557297 26095772

[22] Asada S et al (2018) Mutant ASXL1 cooperates with BAP1 to promote myeloid leukaemogenesis. Nat Commun 9(1):2733 30013160 10.1038/s41467-018-05085-9 PMC6048047

[23] Bai J et al (2021) Reducing hyperactivated BAP1 attenuates mutant ASXL1-driven myeloid malignancies in human haematopoietic cells. Cancer Lett 519:78–90 34186160 10.1016/j.canlet.2021.06.019

[24] Wang L et al (2021) Epigenetic targeted therapy of stabilized BAP1 in ASXL1 gain-of-function mutated leukemia. Nat Cancer 2(5):515–526 35122023 10.1038/s43018-021-00199-4

[25] Pech MF et al (2019) Systematic identification of cancer cell vulnerabilities to natural killer cell-mediated immune surveillance. Elife. 10.7554/eLife.47362 31452512 10.7554/eLife.47362 PMC6713475

[26] Zhuang X, Veltri DP, Long EO (2019) Genome-wide CRISPR screen reveals cancer cell resistance to NK cells induced by NK-derived IFN-γ. Front Immunol 10:2879 31921143 10.3389/fimmu.2019.02879 PMC6917608

[27] Sheffer M et al (2021) Genome-scale screens identify factors regulating tumor cell responses to natural killer cells. Nat Genet 53(8):1196–1206 34253920 10.1038/s41588-021-00889-w

[28] Chiba M et al (2022) Genome-wide CRISPR screens identify CD48 defining susceptibility to NK cytotoxicity in peripheral T-cell lymphomas. Blood 140(18):1951–1963 35921533 10.1182/blood.2022015646 PMC9837448

[29] Dufva O et al (2023) Single-cell functional genomics reveals determinants of sensitivity and resistance to natural killer cells in blood cancers. Immunity 56(12):2816-2835.e13 38091953 10.1016/j.immuni.2023.11.008

[30] Bernareggi D et al (2022) CHMP2A regulates tumor sensitivity to natural killer cell-mediated cytotoxicity. Nat Commun 13(1):1899 35393416 10.1038/s41467-022-29469-0 PMC8990014

[31] Hofman T et al (2024) IFNγ mediates the resistance of tumor cells to distinct NK cell subsets. J Immunother Cancer. 10.1136/jitc-2024-009410 38955423 10.1136/jitc-2024-009410 PMC11218003

[32] Kristenson L et al (2024) Deletion of the *TMEM30A* gene enables leukemic cell evasion of NK cell cytotoxicity. Proc Natl Acad Sci 121(15):e2316447121 38557174 10.1073/pnas.2316447121 PMC11009675

[33] Kandoth C et al (2013) Mutational landscape and significance across 12 major cancer types. Nature 502(7471):333–339 24132290 10.1038/nature12634 PMC3927368

[34] Dey A et al (2012) Loss of the tumor suppressor BAP1 causes myeloid transformation. Science 337(6101):1541–1546 22878500 10.1126/science.1221711 PMC5201002

[35] Andricovich J, Lap CJ, Tzatsos A (2025) Loss of BAP1 defines a unique subtype of TP53-mutated de novo AML and confers sensitivity to BCL-xL inhibitors. Blood 146(12):1493–1510 40540751 10.1182/blood.2024026417 PMC12983020

[36] Xia YK et al (2021) Tumor-derived neomorphic mutations in ASXL1 impairs the BAP1-ASXL1-FOXK1/K2 transcription network. Protein Cell 12(7):557–577 32683582 10.1007/s13238-020-00754-2 PMC8225741

[37] Kurkowiak M et al (2017) Genomic landscape of human erythroleukemia K562 cell line, as determined by next-generation sequencing and cytogenetics. Acta Haematol Pol 48(4):343–349

[38] Bottomly D et al (2022) Integrative analysis of drug response and clinical outcome in acute myeloid leukemia. Cancer Cell 40(8):850-864.e9 35868306 10.1016/j.ccell.2022.07.002 PMC9378589

[39] Wee ZN et al (2014) EZH2-mediated inactivation of IFN-γ-JAK-STAT1 signaling is an effective therapeutic target in MYC-driven prostate cancer. Cell Rep 8(1):204–216 24953652 10.1016/j.celrep.2014.05.045

[40] Burr ML et al (2019) An evolutionarily conserved function of polycomb silences the MHC class I Antigen presentation pathway and enables immune evasion in cancer. Cancer Cell 36(4):385-401.e8 31564637 10.1016/j.ccell.2019.08.008 PMC6876280

[41] Inoue D et al (2016) Truncation mutants of ASXL1 observed in myeloid malignancies are expressed at detectable protein levels. Exp Hematol 44(3):172–6.e1 26700326 10.1016/j.exphem.2015.11.011

[42] LaFave LM et al (2015) Loss of BAP1 function leads to EZH2-dependent transformation. Nat Med 21(11):1344–1349 26437366 10.1038/nm.3947 PMC4636469

[43] Wijdeven RH et al (2024) Balanced epigenetic regulation of MHC class I expression in tumor cells by the histone ubiquitin modifiers BAP1 and PCGF1. J Immunol 212(3):446–454 38088808 10.4049/jimmunol.2300263

[44] Wang B et al (2024) Comprehensive characterization of IFNγ signaling in acute myeloid leukemia reveals prognostic and therapeutic strategies. Nat Commun 15(1):1821 38418901 10.1038/s41467-024-45916-6 PMC10902356

