# Supplementary material for: BAP1 loss impairs IFN-γ signaling and enhances NK cell-mediated cytotoxicity in myeloid leukemia
Source: Cancer Immunol Immunother. 2026 Jun 15;75(6):171. doi: 10.1007/s00262-026-04453-5 (PMC13269568; doi:10.1007/s00262-026-04453-5)
Supplement: Supplementary file 1 — Supplementary file1 (DOCX 5382 KB) [file 262_2026_4453_MOESM1_ESM.docx]

**Supplementary tables**

| **Screen** | **Kristenson et al.** | **Pech et al.** | **Dufva et al.** | **Hofman et al.** | **Bernareggi et al. *** |
| --- | --- | --- | --- | --- | --- |
| **NK cell condition** | Primary | NK-92 | Primary | Primary | Primary |
| **Target cell type** | K562 | K562 | K562 | A375 | Glioblastoma stem cells |
| **BAP1 lfc** | -0.52 | -0.79 | -2.63 | -0.80 |  |
| **BAP1 p-value** | 0.000086 | 0.002 | 0.011 | 0.0001 |  |
| **BAP1 neg rank** | 28 | 107 | 139 |  |  |

**Supplementary Table 1.** BAP1 log fold change (lfc), p-value, and rank (when available) from MAGeCK analysis of CRISPR screens.

* Bernareggi et al. provides a score resulting from 4 CRISPR screens in 4 different GBS cell lines.

| **Gene** | **Sequence** |
| --- | --- |
| *GAPDH* | Forward: CCCACTCCTCCACCTTTGAC  Reverse: GCCAAATTCGTTGTCATACCAGG |
| *TBP* | Forward: CCACTCACAGACTCTCACAAC  Reverse: CTGCGGTACAATCCCAGAACT |
| *BAP1* | Forward: AGGAGCTGCTGGCACTGCTGA  Reverse: AGCATCGGCCGGCTCCACAA |
| *IFNGR1* | Forward: TCCTCAGTGCCTACACCAACTAATG  Reverse: GAGAATGAACGGAAGTGAGATCCAG |
| *HLA-A* | Forward: AGACTGACCGAGTGGACC  Reverse: ACGTCGCAGCCATACATTATC |
| *HLA-B* | Forward: TCCTAGCAGTTGTGGTCATCGG  Reverse: GCTCCCTCCTTTTCCACCTGA |
| *HLA-C* | Forward: CCTGGTTGTCCTAGCTGTCCTT  Reverse: CAGGCTTTACAAGTGATGAGAGACT |
| *HLA-E* | Forward: TAAGGCTGAGTGGAGCGAC  Reverse: AAGACACATAGGGGAGGCGT |

**Supplementary Table 2.** Primers used for qPCR.

**Supplementary figures**

**
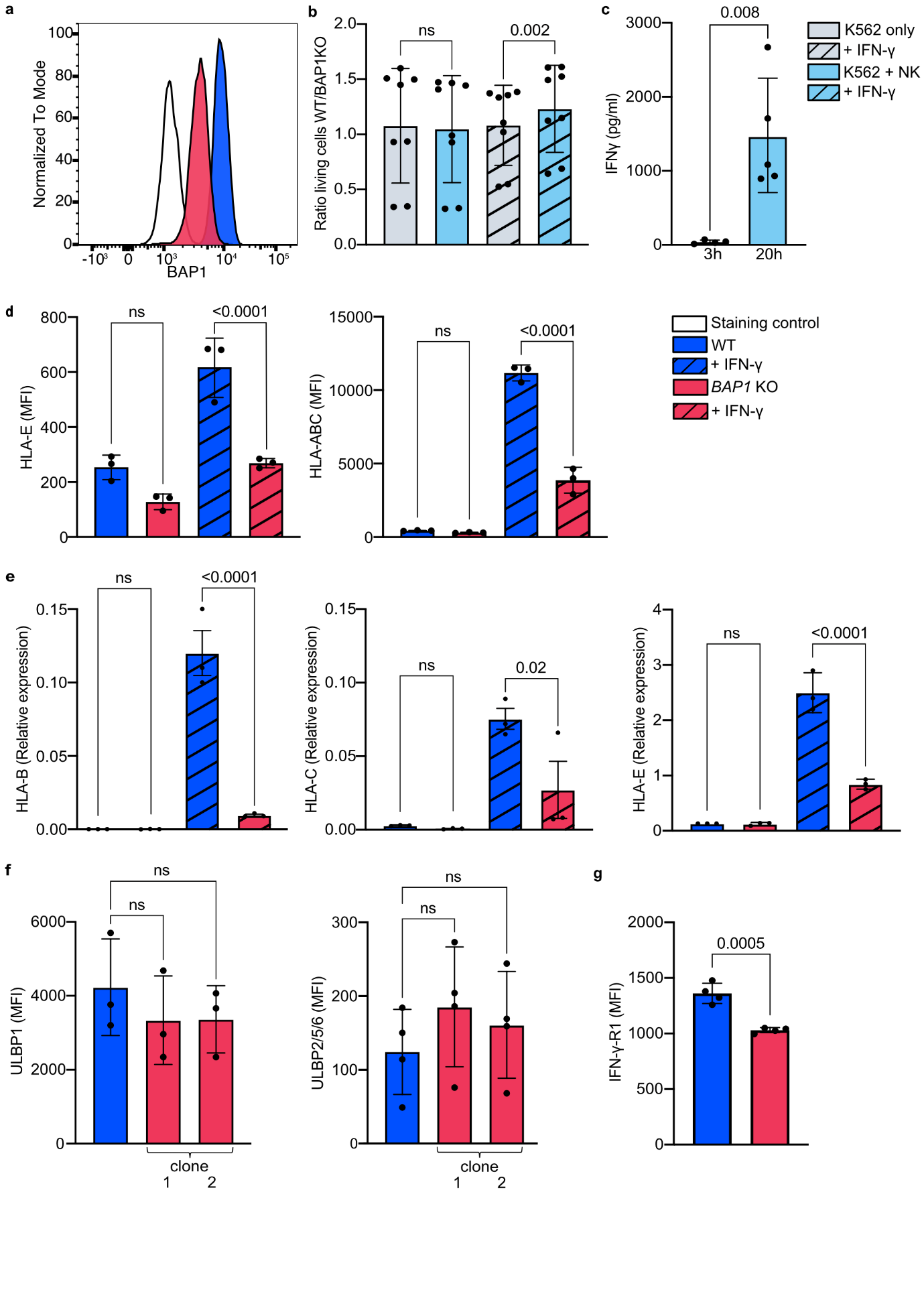
**

**Supplementary Fig. 1**. (a) BAP1 intranuclear staining for WT and *BAP1* KO K562. (b) Dual-target cytotoxicity assay for NK vs untreated and IFN-γ pre-treated *BAP1* KO K562 cells with crRNA 2 vs WT (n=8). (c) IFN-γ ELISA for overnight-activated NK cells co-incubated with WT K562 for 3h (n=4), or polyclonally activated NK cells co-incubated with WT K562 for 20h (n=5). (d) HLA-E and HLA-ABC staining of *BAP1* KO K562 with crRNA 2 vs WT (n=3). (e) HLA-B, HLA-C and HLA-E mRNA expression of *BAP1* KO vs WT K562 (n=3). (f) ULBP1 and ULBP2/5/6 staining of WT vs *BAP1* KO K562. (g) IFN-γ-R1 staining of *BAP1* KO K562 with crRNA 2 vs WT (n=4). One-way Anova followed by Šidák’s multiple comparison test was used in b, d, e and f. t-test was used for statistical analysis in c and g. ns = not significant. Error bars represent SD or SEM.

**
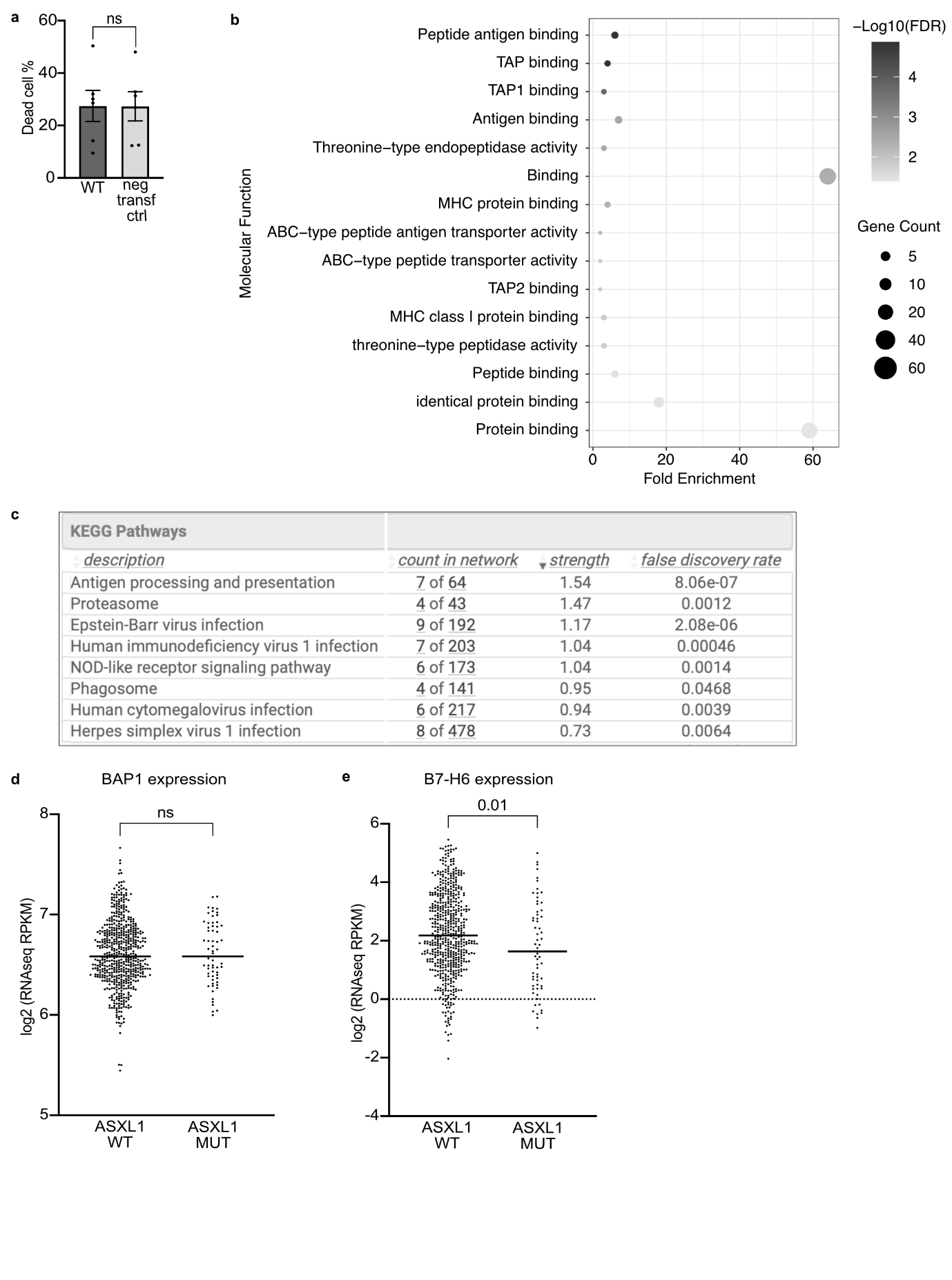
**

**Supplementary Fig. 2.** (a) Death WT and mock-transfected K562 cell percentage after a 3-hour co-incubation with NK cells (n=6). (b-c) Gene ontology molecular function analysis (b) and KEGG pathway analysis (c) from STRING analysis of the downregulated proteins in *BAP1* KO vs WT K562 following IFN-γ treatment that were not downregulated in the untreated condition ranked by strength. (d-e) BAP1 (d) and B7-H6 (e) mRNA expression in AML patients carrying an *ASXL1* mutation (*ASXL1*-MT) or not (*ASXL1*-WT), from OHSU dataset (Bottomly, 2022). t-test was used for statistical analysis in a. Mann-Whitney U-test was used in d and e. ns = not significant. Error bars represent SD.


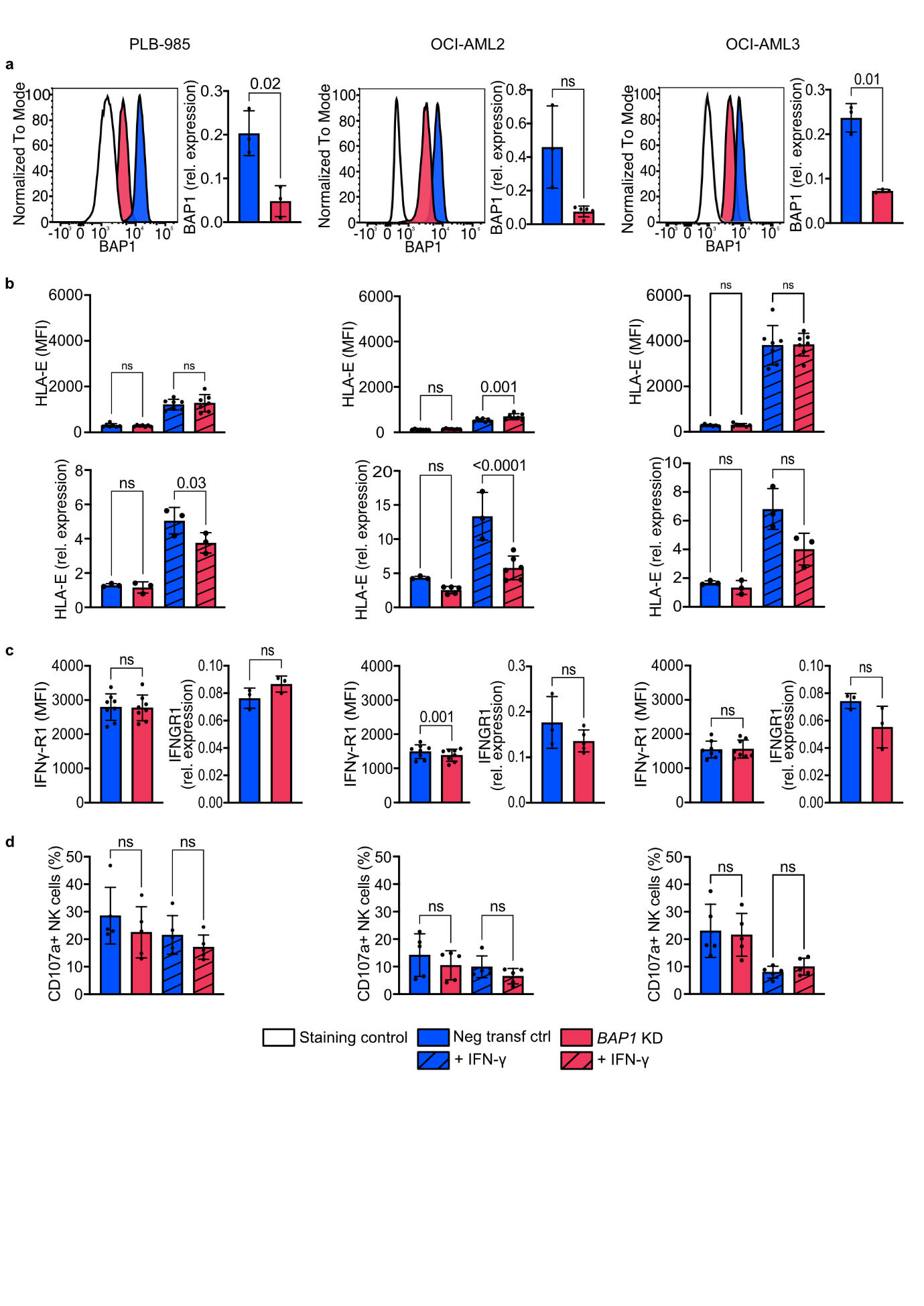


**Supplementary Fig. 3.** (a) Intranuclear BAP1 staining and *BAP1* transcript in *BAP1* KD and negative control-transfected PLB-985, OCI-AML2 and OCI-AML3 cells. (b) HLA-E staining and transcript of *BAP1* KD PLB-985, OCI-AML2 and OCI-AML3 cells compared to negative control without and with IFN-γ treatment. (c) IFN-γ-R1 staining and transcript of *BAP1* KD PLB-985, OCI-AML2 and OCI-AML3 cells compared to negative control without and with IFN-γ treatment. (d) NK degranulation for *BAP1* KD PLB-985, OCI-AML2 and OCI-AML3 cells without and with IFN-γ pre-treatment compared to negative control (n=5). t-test was used for statistical analysis in a and c. One-way Anova and Šidák’s multiple comparison test were used for statistical analysis in b and d. ns = not significant. Error bars represent SD.

**
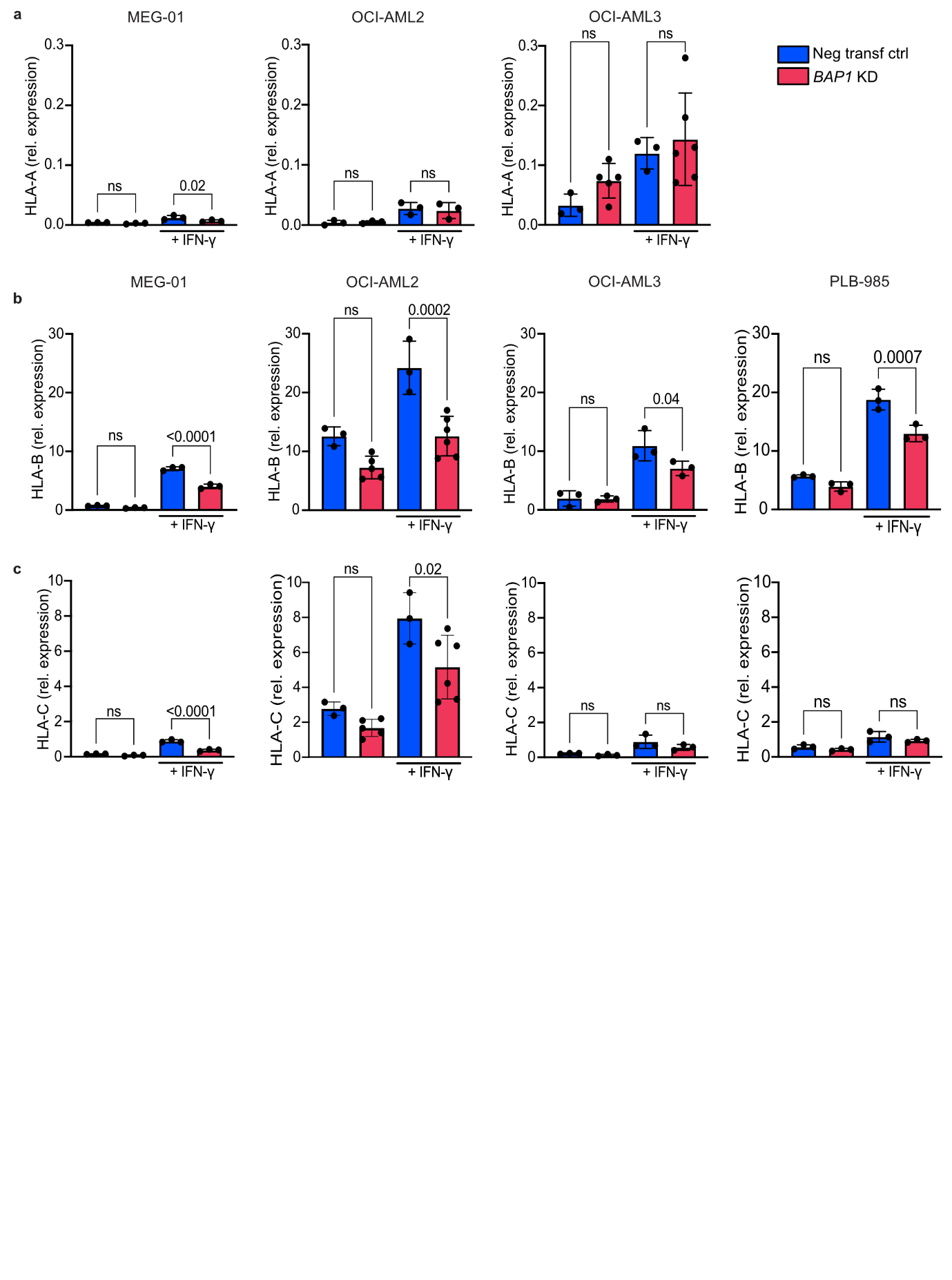
**

**Supplementary Fig. 4**. (a-c) HLA-A (a) transcript of *BAP1* KD vs negative control-transfected MEG-01, OCI-AML2 and OCI-AML3. HLA-B (b) and HLA-C (c) transcripts of *BAP1* KD vs negative control-transfected MEG-01, PLB-985, OCI-AML2 and OCI-AML3. One-way Anova and Šidák’s multiple comparison test were used for statistical analysis in a, b and c. ns = not significant. Error bars represent SD.

**
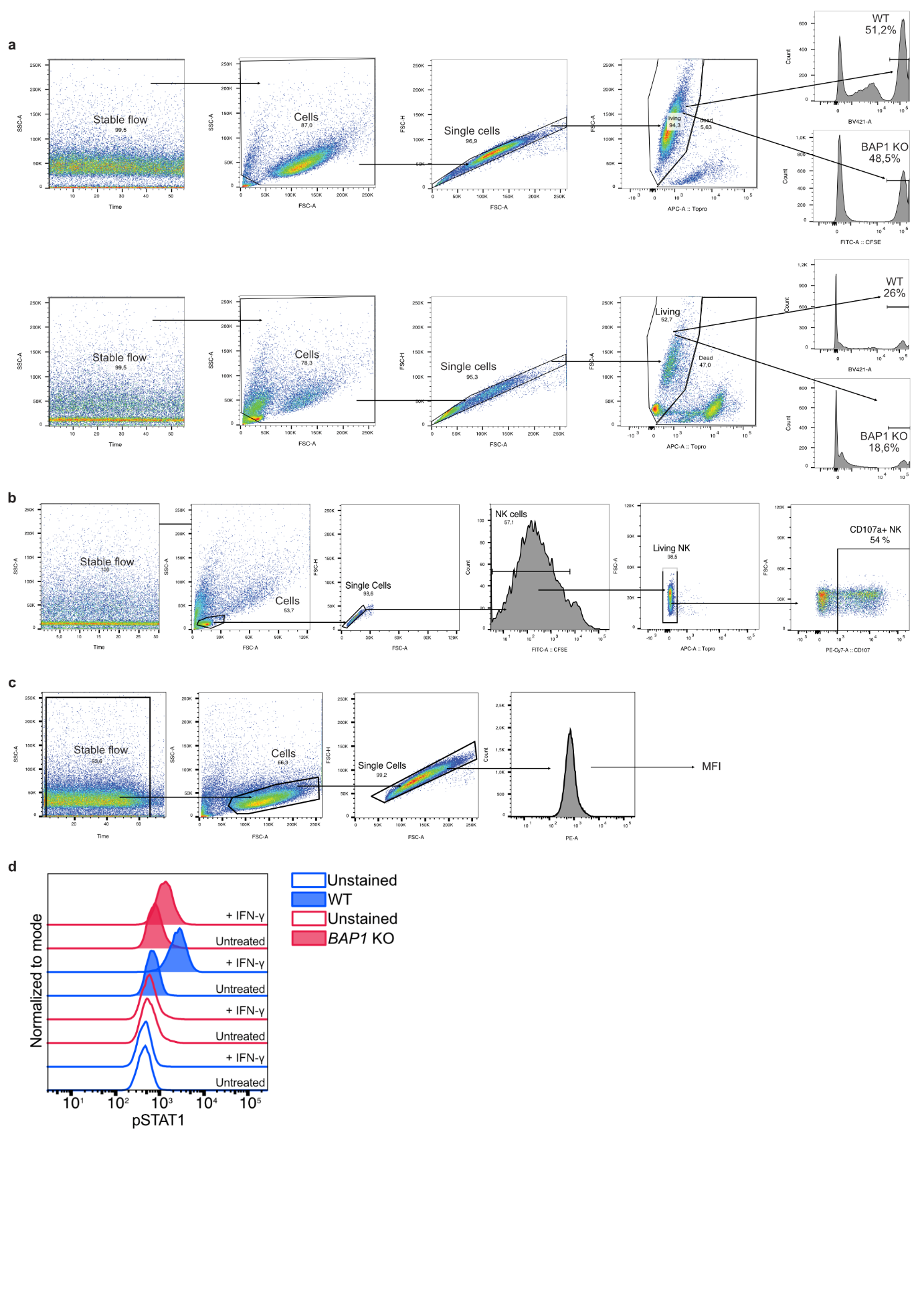
**

**Supplementary Fig. 5.** (a) Gating strategies used for 3-hour dual-labelling cytotoxicity assay. (b) Gating strategy used in 3-hour degranulation assays. (c) Gating strategy used in staining. (d) Representative histogram for pSTAT1 staining.
